# Supplementary material for: Quantification reveals early dynamics in Drosophila maternal gradients
Source: PLoS One. 2021 Aug 19;16(8):e0244701. doi: 10.1371/journal.pone.0244701 (PMC8376041; doi:10.1371/journal.pone.0244701)
Supplement: S1 File — (DOCX) [file pone.0244701.s001.docx]

Quantification reveals early dynamics in *Drosophila* maternal gradients

Alex Shlemov,^1^ Theodore Alexandrov,^2,3^ Nina Golyandina,^4^ David Holloway,^5^ Stefan Baumgartner,^6,7^ Alexander V. Spirov^8,9,‡^

^1^Laboratory for Algorithmic Biology, St.Petersburg State University, St.Petersburg, Russia

^2^Structural and Computational Biology Unit, European Molecular Biology Laboratory, Heidelberg, Germany

^3^Skaggs School of Pharmacy and Pharmaceutical Sciences, University of California San Diego, CA, La Jolla, USA

^4^Faculty of Mathematics and Mechanics, St. Petersburg State University, St.Petersburg, Russia

^5^Mathematics Department, British Columbia Institute of Technology, Burnaby, BC, Canada

^6^Department of Experimental Medical Sciences, Lund University, Lund, Sweden

^7^Department of Biology, University of Konstanz, Konstanz, Germany

^8^Computer Science and CEWIT, SUNY Stony Brook, Stony Brook, NY, USA

^9^Lab Modelling Evolution, The I.M. Sechenov Institute of Evolutionary Physiology & Biochemistry, St.Petersburg Russia

^‡^Corresponding author, [alexander.spirov@gmail.com](mailto:alexander.spirov@gmail.com)

# SUPPORTING INFORMATION

S1 Table. Results of assignment of embryos to the three development stages using Linear Discriminant Analysis for the bcd mRNA profiles.

|  | **Percent - Correct** | **cleavage** | **st1013** | **st14** |
| --- | --- | --- | --- | --- |
| **cleavage** | 70 | 14 | 4 | 2 |
| **st1013** | 76 | 5 | 19 | 1 |
| **st14** | 79 | 4 | 6 | 37 |

S2 Table. Results of assignment of embryos to the three development stages using Linear Discriminant Analysis for the Staufen factor profiles.

|  | **Percent - Correct** | **cleavage** | **st1013** | **st14** |
| --- | --- | --- | --- | --- |
| **cleavage** | 50 | 3 | 2 | 1 |
| **st1013** | 73 | 2 | 11 | 2 |
| **st14** | 69 | 2 | 2 | 9 |

S3 Table. The mean parameter sets for three developmental stages for the bicoid mRNA profiles.

| Bicoid |  |  |  |  |  |
| --- | --- | --- | --- | --- | --- |
| group3 | 1lam1prc | 1lam2prc | 2lam1prc | 2lam2prc | C1_prof12 |
| cleavage | 0.825770 | 0.996955 | 0.837744 | 0.996547 | 0.274277 |
| st1013 | 0.880132 | 0.993494 | 0.855254 | 0.993852 | -0.16707 |
| st14 | 0.872287 | 0.993154 | 0.836638 | 0.993174 | 0.657256 |

S4 Table. The mean parameter sets for three developmental stages for the Staufen factor profiles.

| Staufen |  |  |  |  |  |
| --- | --- | --- | --- | --- | --- |
| stage3 | 1lam1prc | 1lam2prc | 2lam1prc | 2lam2prc | c1_prof12 |
| cleavage | 0.793801 | 0.999038 | 0.819559 | 0.998701 | -0.14991 |
| st1013 | 0.870181 | 1.000127 | 0.867264 | 0.999466 | -0.27331 |
| st14 | 0.861620 | 0.999261 | 0.890012 | 1.000570 | 0.408883 |

**References**

Shlemov A, Golyandina N, Holloway D, Spirov A. Shaped singular spectrum analysis for quantifying gene expression, with application to the early Drosophila embryo. Biomed Res Int. 2015;2015:689745. doi: 10.1155/2015/689745.

Little SC, Tkacik G, Kneeland TB, Wieschaus EF, and Gregor T (2011) The Formation of the Bicoid Morphogen Gradient Requires Protein Movement from Anteriorly Localized mRNA. PLoS Biol 9(3): e1000596. doi: 10.1371/journal.pbio.1000596.
