# Supplementary figures and images for: Quantification reveals early dynamics in Drosophila maternal gradients
Source: PLoS One. 2021 Aug 19;16(8):e0244701. doi: 10.1371/journal.pone.0244701 (PMC8376041; doi:10.1371/journal.pone.0244701)

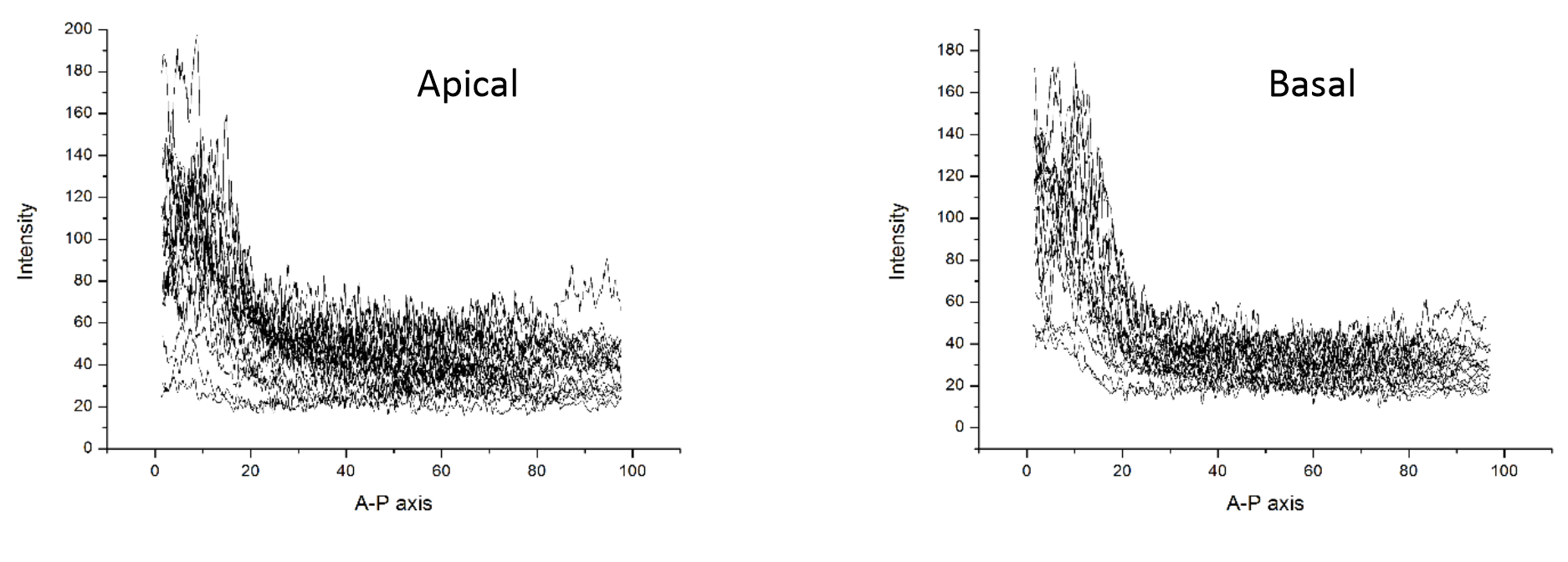

Supplement: S1 Fig — (TIF) [file pone.0244701.s002.tif]

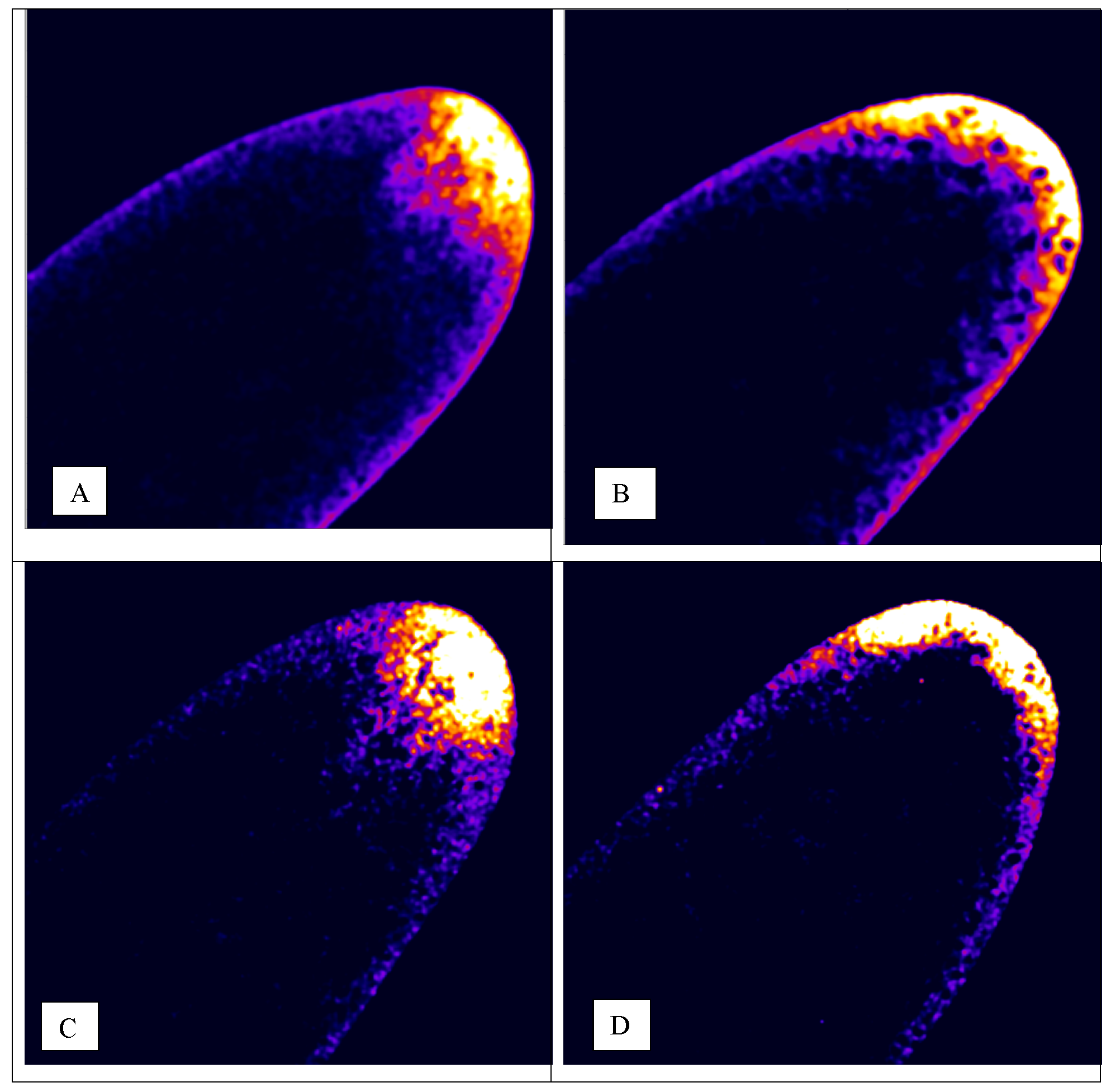

Supplement: S2 Fig — (TIF) [file pone.0244701.s003.tif]

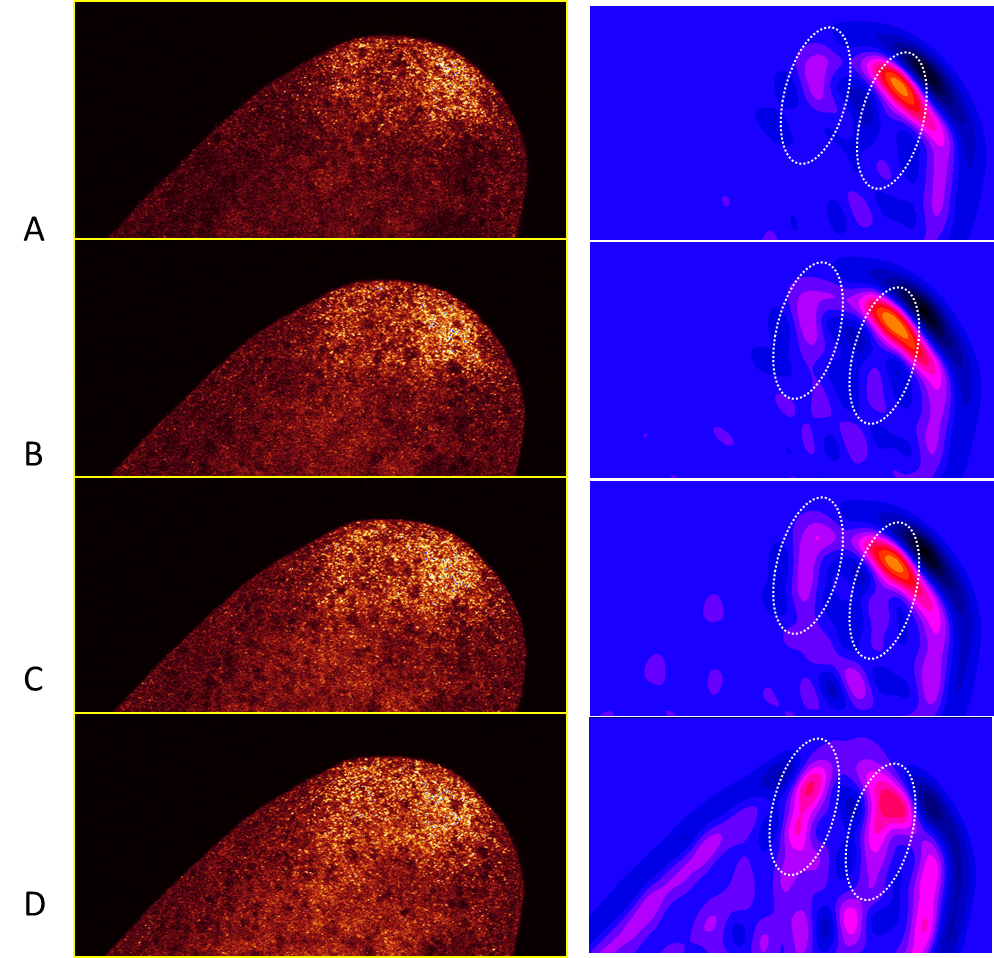

Supplement: S3 Fig — (TIF) [file pone.0244701.s004.tif]

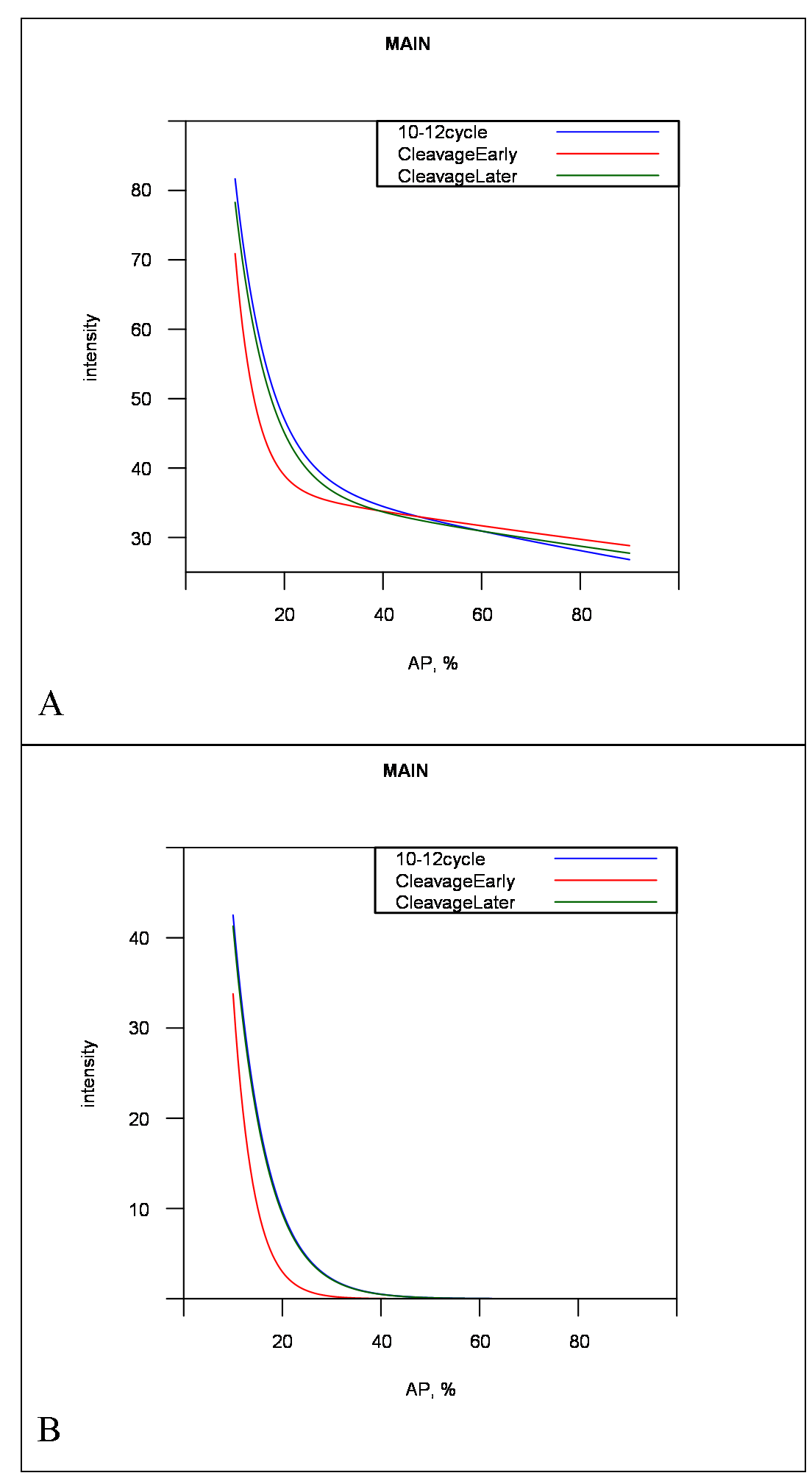

Supplement: S4 Fig — (TIF) [file pone.0244701.s005.tif]

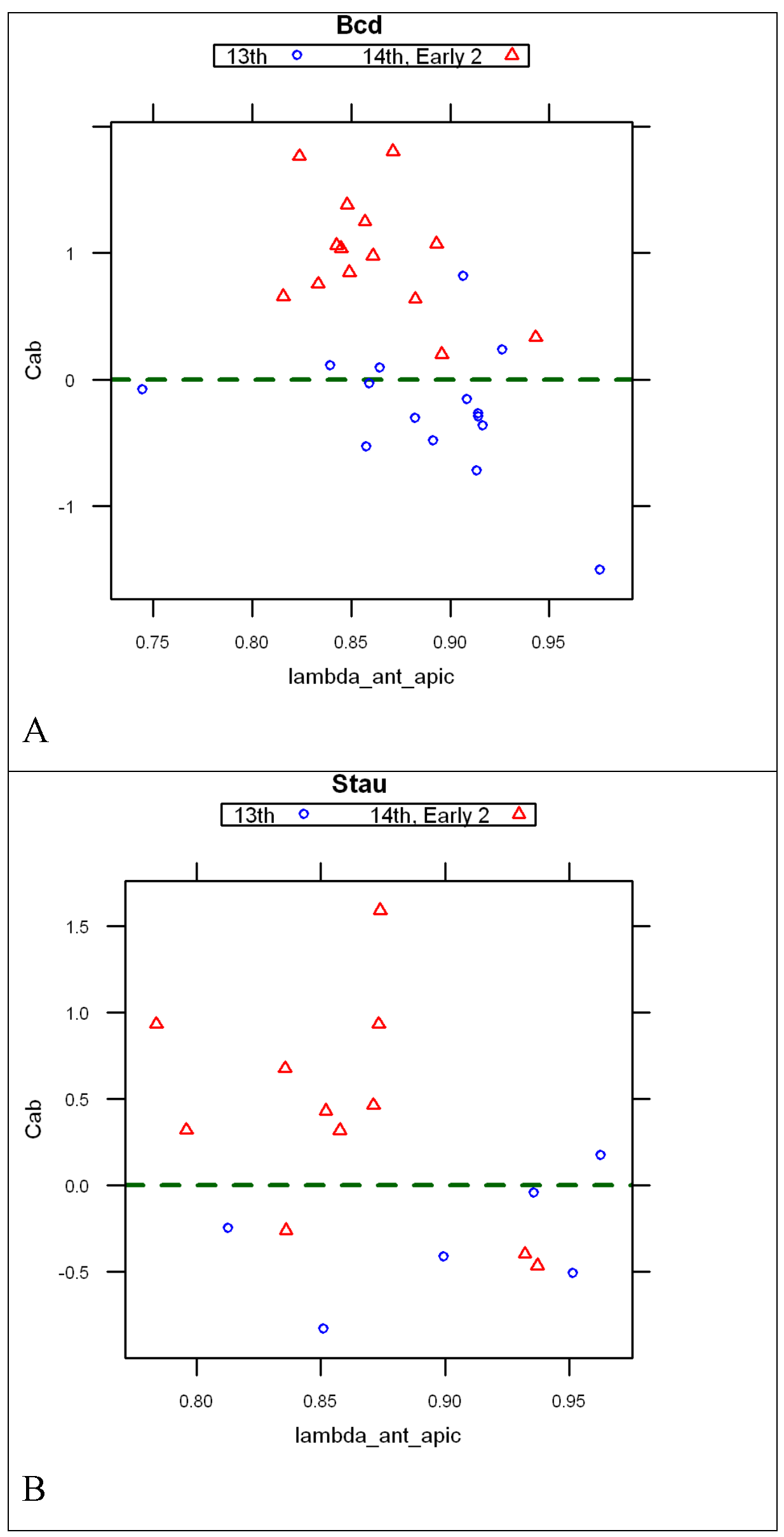

Supplement: S5 Fig — (TIF) [file pone.0244701.s006.tif]
