# Supplementary material for: Quantification reveals early dynamics in Drosophila maternal gradients
Source: PLoS One. 2021 Aug 19;16(8):e0244701. doi: 10.1371/journal.pone.0244701 (PMC8376041; doi:10.1371/journal.pone.0244701)
Supplement: Attachment — Submitted filename: SpirovResponse-Jul2021.docx [file pone.0244701.s008.docx]

Dear Dr. Schubert,

Thank you very much for your and the reviewers’ work on reading our revised submission. We are glad that Reviewers 2 and 3 are satisfied with the revisions and feel that the work is ready for publication. We respond to Reviewer 1’s specific comments in this letter. There don’t appear to be indications for further revision of the manuscript itself, but we can do so if there are specific indications.

*6. Review Comments to the Author*

*Reviewer #1: Fortunately, the authors eliminated from the introduction the reference to the French flag model.*

**Done.**

*There are statements in the abstract that are known from the time of Crick (1960). For example: “supporting that the distribution and dynamics of bcd mRNA are key factors in the formation and maintenance of the Bcd protein morphogenetic gradient”. This is the so called “central dogma of molecular genetics”.*

**This section was not to revisit the central dogma, but rather to highlight that Bcd protein gradient formation has been frequently studied in isolation from *bcd* mRNA spatial patterning or the temporal dynamics of the *bcd* mRNA.**

*The statement “The observed co-localization during redistribution of bcd and Stau may indicate the involvement of active transport.”. If there is active transport, the equations describing the distribution are not exponential. By the way, the exponential function is only compatible with a diffusion mechanism (not even reaction-diffusion).*

**As mentioned below, the exponential fit is phenomenological.**

*It is now clear that the so called two or three exponential equation is a phenomenological fit and the analysis presented by the authors is irrelevant to validate biological mechanisms. Fits are not models and are not used for validation. A mathematical model passes by 3 stages: benchmarking, calibration and validation. None of this has been done*.

**Correct, this study quantifies the data. This suggests the traditional SDD mathematical model is inadequate, but there is no new mathematical modelling involved. New models should be able to account for the dynamic shifts we report. The mathematical aspects of the (phenomenological) model are discussed, among other things, and the results are justified in [Alexandrov T., Golyandina N., Holloway D., Shlemov A., and Spirov A. 2018. Two-exponential models of gene expression patterns for noisy experimental data. J.Comp.Biol. 25:1220-1230]. (This ref is from the paper, ref [60], not new.)**

*The exponential functions do not justify the profiles of Bcd, cf., for example, figure 2a, of Houchmandzadeh, Wieschaus, Leibler, Nature 415 (2002) 798-802. Moreover, there is a large variability found in the tails of BCD in different individuals. This analysis has not been done for bcd.*

**Bcd protein profiles are not analyzed in this paper. As discussed above, exponentials are the basis functions for the phenomenological quantification of the *bcd* mRNA data. Variability is apparent and addressed across n = 160 *bcd* profiles, e.g. in Figs. 3, 7, 10, 11, 13.**

*I have some difficulty in extracting useful biological information from this paper. Anyhow, I recognize the strong effort of some of the biological oriented authors. I think that there are some misinterpretations about the role of mathematical models in biology.*

**This is a quantification of the *bcd* mRNA and Stau protein gradient data.**

Please let us know if there are any further questions.

With best regards,

Alexander Spirov
